# Evidence of scrapie transmission via milk

**DOI:** 10.1186/1746-6148-4-14

**Published:** 2008-04-08

**Authors:** Timm Konold, S Jo Moore, Susan J Bellworthy, Hugh A Simmons

**Affiliations:** 1 Neuropathology, Veterinary Laboratories Agency Weybridge, New Haw, Addlestone, Surrey KT15 3NB, UK; 2 Population Biology and Disease Control Research Group, Royal Veterinary College, North Mymms, Hatfield, Hertfordshire AL9 7TA, UK; 3 Animal Services Unit, Veterinary Laboratories Agency Weybridge, New Haw, Addlestone, Surrey KT15 3NB, UK

## Abstract

**Background:**

The risk of scrapie infection increases with increased duration and proximity of contact between sheep at lambing. Scrapie infectivity has not been detected in milk but cellular prion protein, the precursor of disease-associated prion protein PrP^d^, has been found in milk from ruminants. To determine whether milk is able to transmit scrapie, 18 lambs with a prion protein genotype associated with high susceptibility to scrapie (VRQ/VRQ) were fed milk from twelve scrapie-affected ewes of the same genotype, and 15 VRQ/VRQ sheep reared on scrapie-free dams served as controls.

**Results:**

Three lambs fed milk from scrapie-affected ewes were culled due to intercurrent diseases at 43, 44 and 105 days of age respectively, and PrP^d ^was detected in the distal ileum of the first two lambs, whilst PrP^d ^was not found in lymphoreticular tissues in the third lamb. A control lamb, housed in a separate pen and culled at 38 days of age, was also negative for PrP^d ^in a range of tissues. Samples of recto-anal mucosa associated lymphoid tissue collected from the remaining 15 live lambs at seven months of age (between five to seven months after mixing) were positive for PrP^d ^in the scrapie milk recipients, whereas PrP^d ^was not detected in the remaining 14 controls at that time. A subsequent sample collected from control lambs revealed PrP^d ^accumulation in two of five lambs eight months after mixing with scrapie milk recipients suggestive of an early stage of infection via lateral transmission. By contrast, the control sheep housed in the same building but not mixed with the scrapie milk recipients were still negative for PrP^d^.

**Conclusion:**

The presence of PrP^d ^in distal ileum and rectal mucosa indicates transmission of scrapie from ewe to lamb via milk (or colostrum) although it is not yet clear if such cases would go on to develop clinical disease. The high level of infection in scrapie-milk recipients revealed by rectal mucosal testing at approximately seven months of age may be enhanced or supplemented by intra-recipient infection as these lambs were mixed together after feeding with milk from scrapie-affected ewes and we also observed lateral transmission from these animals to lambs weaned from scrapie-free ewes.

## Background

Scrapie is a neurological disease in sheep, which belongs to the group of transmissible spongiform encephalopathies (TSEs) or prion diseases. The disease is usually confirmed by the detection of the disease-associated prion protein, PrP^d^, in lymphoreticular tissues and tissues of the central nervous system.

Studies on the pathogenesis of natural scrapie in sheep have demonstrated that PrP^d ^is first detected in the lymphoid tissues of the digestive tract at two months of age [1,2], which suggests that infection occurs *in utero *or soon after birth. The oral route is consistent with an epidemiological study, which demonstrated that the risk of infection increased with increased duration and proximity of contact between sheep at lambing [3]. The main source of infection is believed to be the placenta as this tissue contains infectivity [4] and PrP^d ^[5]. Other possible sources are faeces, saliva, urine, colostrum, milk or blood [6]. Parenteral infection of mice with colostrum or milk from sheep affected by scrapie [4,7] or goats with milk from a scrapie-affected goat [8] has not produced disease. More recently, milk from mice expressing bovine PrP, which were intracerebrally infected with bovine spongiform encephalopathy (BSE) prions, also failed to transmit disease experimentally in the same species [9]. These findings suggest that milk may not be important in the transmission of infectivity, although such extrapolation to natural infections in small ruminants may not be appropriate. Cellular prion protein, which transforms into PrP^d ^in prion disease, is found in the milk from ruminants therefore milk from an infected animal could serve as a source for PrP^d ^and even transmit disease [10]. In addition, there is evidence that maternal milk could be responsible for transmission as the risk of scrapie is reduced when lambs are reared on artificial milk after ingestion of the dam's colostrum compared to lambs that are entirely maternally fed [11]. The objective of this study was to investigate the risk of milk as a source of scrapie infection by feeding milk from scrapie-affected ewes to lambs.

## Results

### Lambs

Four lambs were culled as a result of intercurrent diseases: three lambs, two from a pair of lambs fed milk from different scrapie-affected ewes, were culled at 43 (lamb number 517/7), 44 (lamb number 516/7) and 105 days (lamb number 518/7) respectively. One building control lamb (number 548/7) was culled at 38 days of age.

The remaining lambs (15 scrapie milk recipients, nine building controls and five lateral transmission controls are currently alive at approximately eleven months of age (as at February 2008).

### PrP^d ^test results

#### Culled lambs

**Figure 1:**
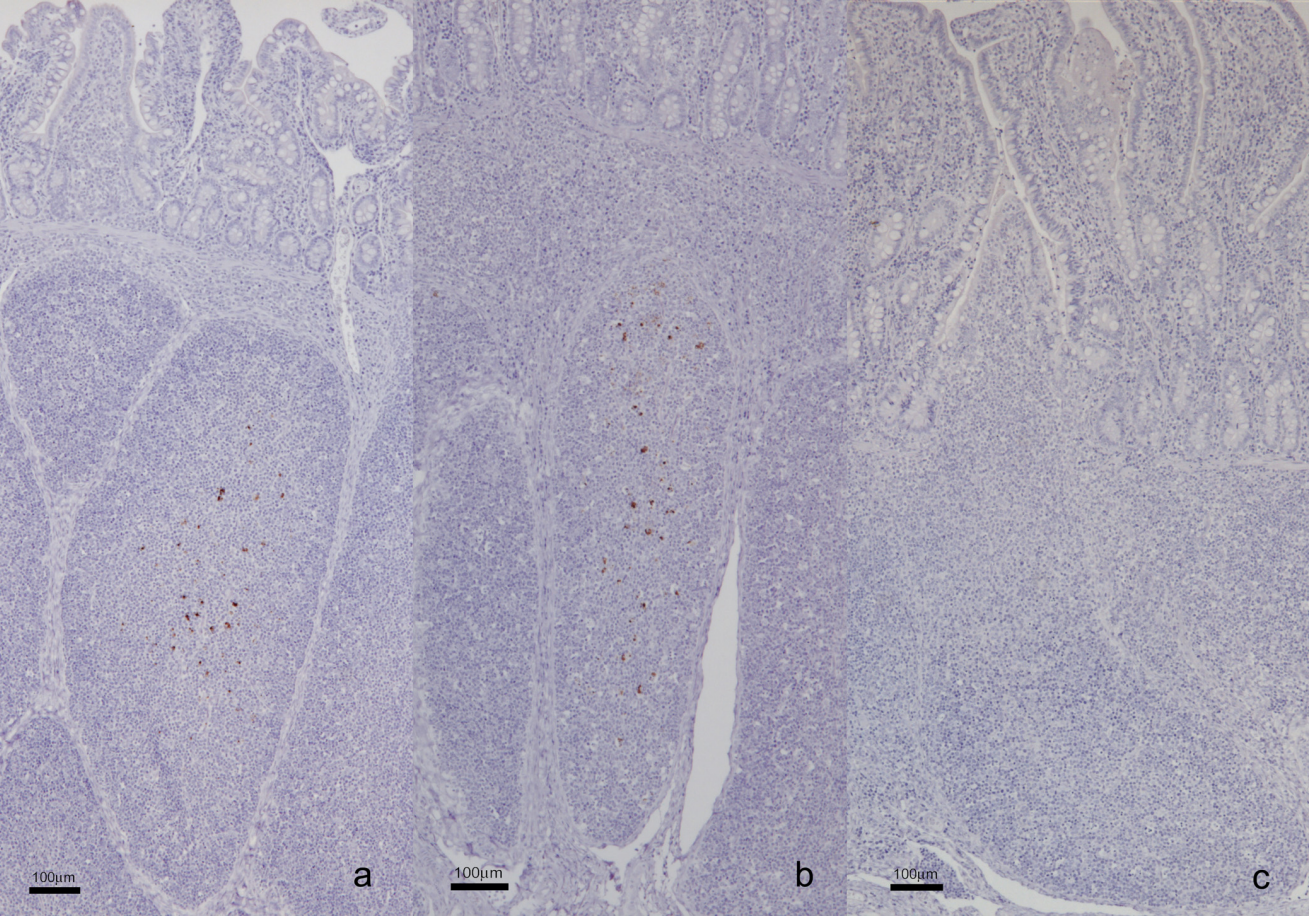
**Immunolabelling of a section of the distal ileum containing Peyer's patches with PrP antibody R145**. a-b) PrP^d ^accumulation is visible in lymphoid follicles of two lambs (a = 516/7, b = 517/7) fed milk from scrapie-affected ewes. c) PrP^d ^immunolabelling is absent in lymphoid follicles of  the control lamb (c = 548/7) reared by its TSE-free dam. Scale bars, 100 μm.

Immunohistochemical examination of the distal ileum (Peyer's patches) of two lambs (numbers 516/7 and 517/7) fed milk from scrapie-affected ewes revealed accumulation of PrP^d ^in the lymphoid follicles (see Figures 1a–b). In contrast, PrP^d ^immunolabelling was absent in the distal ileum of lamb number 518/7, fed milk from another scrapie-affected ewe, and in the building control lamb number 548/7 (see Figure 1c). The number of visible lymphoid follicles in a section of the distal ileum was less in lamb 518/7 (36 follicles in total, even after processing of three different sections) compared to the other lambs (97–547 follicles). Disease-associated PrP was not detected by immunohistochemistry (IHC) and Western immunoblot (WB) in the brainstem samples from all four lambs.

#### Remaining live lambs

**Figure 2:**
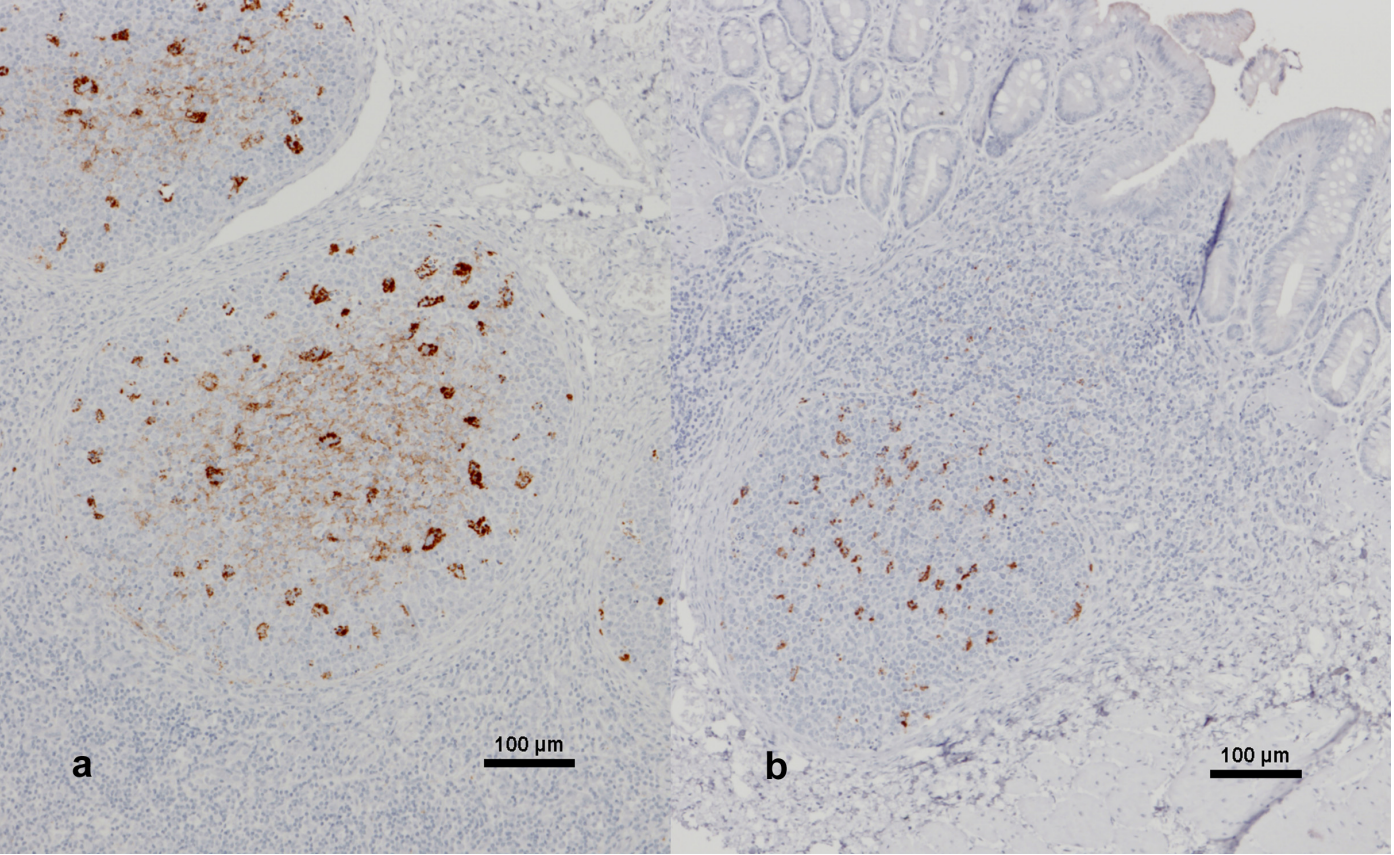
**Immunolabelling of a section of RAMALT with PrP antibody R145**. a) Scrapie milk recipient. PrP^d ^immunolabelling is evident in TBM and FDC (diffuse immunolabelling in the centre) of the lymphoid follicles of the rectal mucosa in lamb 07–1097, which was the partner of 517/7 and also received milk from ewe 1010/6. b) Lateral transmission control. PrP^d ^immunolabelling is confined to TBM only in lymphoid follicles of the rectal mucosa in sheep 07–1109, which was mixed with scrapie milk recipients eight months earlier. Scale bars, 100 μm.

At approximately seven months of age, all samples of recto-anal mucosa associated lymphoid tissue (RAMALT) from the scrapie milk recipients, including the partner lambs of the lambs 516/7 and 517/7 which were culled early in the study, contained disease-associated PrP. PrP^d ^was present in most or all of the lymphoid follicles of the examined section, with both tingible body macrophages (TBM) and moderate to marked follicular dendritic cell (FDC) immunolabelling (see Figure 2a). In one scrapie milk recipient, immunolabelling was confined to TBM, and only in a minority of follicles. The severity of labelling observed did not appear to be related to age at mixing. By contrast, none of the control lambs had detectable PrP^d ^in the RAMALT sample at that time, although in one building control lamb the examined section contained only one lymphoid follicle. The second biopsy sample collected from the control lambs approximately three months later showed PrP^d ^in two of five lateral transmission controls. Immunolabelling in these two controls was confined to TBM only (see Figure 2b), and only up to half the follicles were positive. Two building controls did not have lymphoid follicles in the examined section; the remaining seven lambs did not show PrP^d ^in the lymphoid follicles of RAMALT. Table 1 displays the RAMALT test results in relation to age and mixing of the lambs.

**Table 1:** RAMALT test results in scrapie milk recipients and lateral transmission controls and timing of testing in relation to age and mixing

**Animal number**	**RAMALT Diagnosis**	**Immuno-labelling**		**Time [days] between**			
		
		**TBM**	**FDC**	**Birth and mixing**	**Mixing and 1^st ^RAMALT test**	**Birth and 1^st ^RAMALT test/death**	**Mixing and 2^nd ^RAMALT test**
***Scrapie milk recipients – median***				***16***	***199***	***232***	

07–1359	Positive	Yes	Yes	8	199	207	Not done
07–1360	Positive	Yes	Yes	8	199	207	Not done
518/7	Negative	No	No	8	Not done*	105	Not done
07–1362	Positive	Yes	Yes	8	199	207	Not done
07–1357	Positive	Yes	No	9	199	208	Not done
07–1358	Positive	Yes	Yes	9	199	208	Not done
07–1097	Positive	Yes	Yes	13	219	232	Not done
517/7	Not done			13	Not done	43	Not done
07–1089	Positive	Yes	Yes	16	219	235	Not done
07–1090	Positive	Yes	Yes	16	219	235	Not done
07–1095	Positive	Yes	Yes	19	213	232	Not done
07–1096	Positive	Yes	Yes	19	213	232	Not done
516/7	Not done			20	Not done	44	Not done
07–1094	Positive	Yes	Yes	20	213	233	Not done
07–1091	Positive	Yes	Yes	34	199	233	Not done
07–1092	Positive	Yes	Yes	34	199	233	Not done
07–1099	Positive	Yes	Yes	42	190	232	Not done
07–1252	Positive	Yes	Yes	42	190	232	Not done

***Lateral transmission controls – median***				***72***	***162***	***234***	***242***

07–1109	Positive	Yes	No	70	162	232	242
07–1108	Negative	No	No	71	162	233	242
07–1107	Positive	Yes	No	72	162	234	242
07–1245	Negative	No	No	112	122	234	202
07–1246	Negative	No	No	112	122	234	202

* RAMALT examined at cull, 97 days after mixing

### Milk donor findings

Table 2 displays the breed of each scrapie-affected ewe, the milk volume that was collected and subsequently fed to lambs, the start and length of lactation, the age at cull and the lactation stage when definite clinical signs of scrapie were observed.

**Table 2:** Milk donor details

**Dam ID**	**Breed**	**Number of recipient lambs fed**	**Total volume collected/fed [kg]**	Start of lactation, days of lactation, age at cull*	**Definite clinical signs observed at**
140/6	Cheviot	1	5	21 m 24 d, 13 d, 22 m 15 d	Start of lactation
139/6	PD × Fries	1^518/7(-)^	6.5	21 m 26 d, 12 d, 22 m 12 d	Start of lactation
326/7	PD	1	8	21 m 14 d, 22 d, 22 m 26 d	End of lactation
695/7	PD × Fries	1	8	21 m 17 d, 23 d, 27 m 21 d	End of lactation
352/7	PD	1	10.5	43 m 5 d, 20 d, 47 m 6 d	Mid lactation
350/7	PD	1	11.5	21 m 14 d, 23 d, 23 m 1 d	Start of lactation
1010/6	PD	2^517/7(+)^	39	43 m 5 d, 39 d, 45 m 4 d	Mid lactation
528/7	PD	2	42	19 m 2 d, 51 d, 25 m 14 d	End of lactation
1011/6	PD	2	60.5	43 m 4 d, 52 d, 45 m 17 d	Mid lactation
54/6	PD	2^516/7(+)^	70.5	32 m 10 d, 54 d, 38 m 27 d	Mid lactation
225/6	PD	2	76	21 m 28 d, 45 d, 23 m 6 d	Mid lactation
55/6	PD	2	121	32 m 7 d, 71 d, 38 m 25 d	End of lactation

* Months are displayed in calendar months

PD Poll Dorset

Fries Friesland

^518/7(-) ^Culled lamb without detectable PrP^d^

^516/7(+) ^Culled lamb with detectable PrP^d^

^517/7(+) ^Culled lamb with detectable PrP^d^

**Figure 3:**
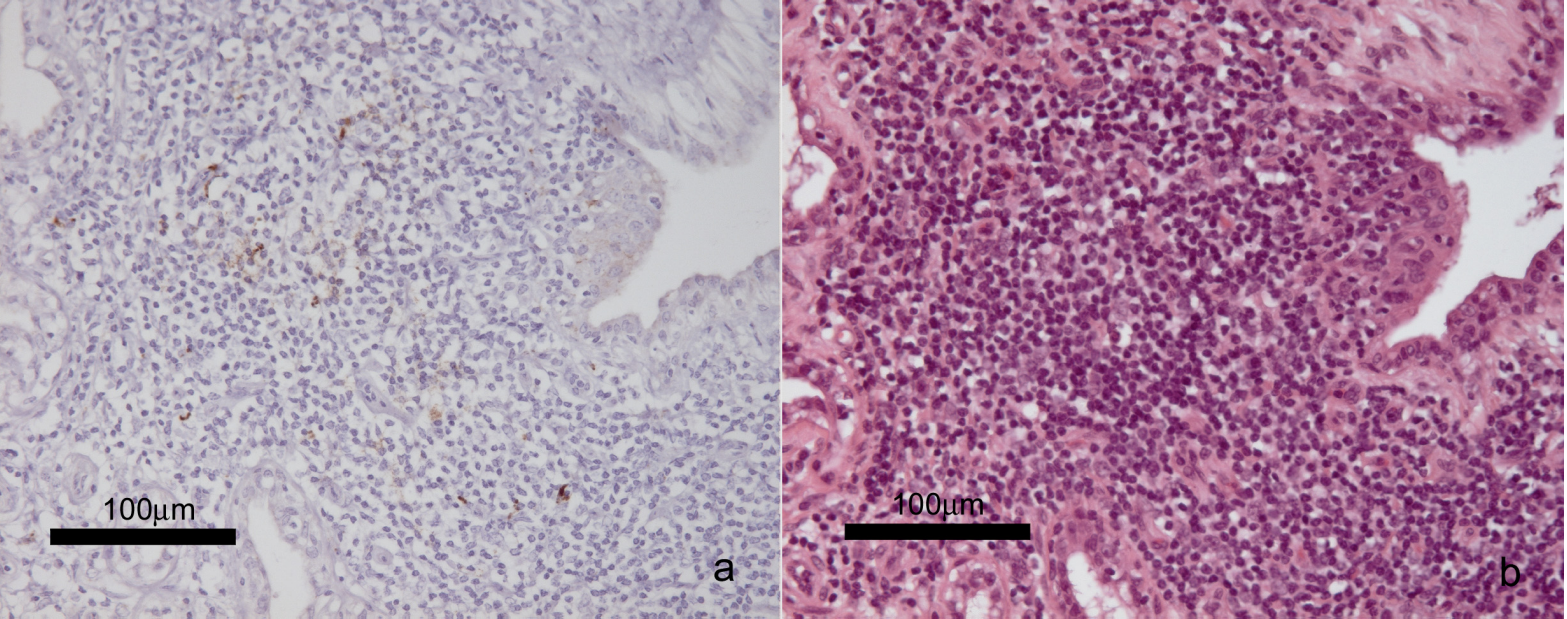
**Lymphoid aggregates in the mammary gland of ewe 1010/6 with accumulation of PrP^d^**. a) PrP^d ^immunolabelling with PrP antibody R145 in lymphoid aggregates within the mammary gland. b) The same section of the mammary gland, stained with H&E, showing the aggregated lymphoid cells. Scale bars, 100 μm.

The ewes that provided the milk for the two artificially reared, PrP^d^-positive lambs that were culled with incidental diseases presented with PrP^d^-immunolabelling in the superficial inguinal lymph nodes. Lymphoid aggregates in the mammary gland were only visible in ewe 1010/6, and PrP^d ^was present in three of six aggregates (see Figure 3).

The ewe (139/6) that provided the milk for the PrP^d^-negative lamb culled early in the study also presented with PrP^d ^accumulation in the superficial inguinal lymph node but PrP^d ^was not found in the single lymphoid aggregate present in the section of the mammary gland.

The somatic cell count (SCC) in a milk sample collected each week from the ewes is listed in Table 3.

**Table 3:** Somatic cell count in weekly milk samples from the ewes

**Ewe – animal number**											
**140/6**	139/6*	**326/7**	**695/7**	**350/7**	**352/7**	**1010/6**	**528/7**	**1011/6**	**225/6**	**54/6**	**55/6**
122 (8)	746 (9)	109 (7)	123 (8)	237 (8)	68 (8)	987 (8)	15 (11)	23 (9)	67 (10)	72 (7)	26 (8)
		33 (14)	38 (15)	49 (15)	73 (15)	370 (15)	10 (18)	98 (16)	17 (17)	22 (14)	45 (15)
					259 (22)	3,495 (22)	20 (25)	15 (23)	8 (24)	35 (21)	23 (22)
						4,088 (29)	30 (32)	90 (30)	17 (31)	42 (28)	15 (29)
						1,545 (36)	1,333 (39)	95 (37)	20 (38)	31 (35)	9 (36)
							17 (45)	45 (44)	32 (45)	50 (42)	20 (43)
										29 (49)	14 (50)
										373 (56)	27 (57)
											41 (64)
											32 (71)

The SCC is displayed in 10^3 ^cells/ml and followed by the day of lactation when the milk sample was collected, in parentheses.

* Ewe that fed lamb with no detectable PrP^d ^when culled.

## Discussion

In naturally infected sheep of highly susceptible genotypes (including VRQ/VRQ), PrP^d ^is associated with early infection with scrapie [1,2,12] and is generally detected in the lymphoreticular system prior to spread and accumulation in the central nervous system and development of clinical disease. We concluded that the two lambs with detectable PrP^d ^in the distal ileum were infected with scrapie and that the source of infection was the milk from scrapie-affected ewes. Both ewes developed definite neurological signs of scrapie during the lactation. Immunohistochemical examination of RAMALT samples collected from the remaining recipient lambs at seven months of age showed PrP^d ^accumulation in all lambs, including the partner lambs of the two culled, PrP^d^-positive animals. The absence of detectable PrP^d ^in the distal ileum of the building control lamb, which was culled early in the experiment, and in the RAMALT samples from the remaining building controls collected twice within an interval of three months, supported our conclusion that environmental contamination was not responsible for infection with the scrapie agent. PrP^d ^was also detected in the remaining live scrapie recipients at approximately seven months of age, at a median time of 199 days after mixing. The first RAMALT biopsy in the lateral transmission controls was taken at a median time of 162 days after introduction to the scrapie milk recipients and found no evidence of infection via lateral transmission at that time. The second sample collected at a median time of 242 days after mixing of controls with scrapie milk recipients (at least equivalent to the time between mixing of scrapie milk recipients and first biopsy) revealed that lateral transmission had already occurred in two of the five controls. This could imply that some of the scrapie milk recipients were in fact infected via lateral transmission from other PrP^d^-positive scrapie-milk recipients, such as the lambs culled early in the experiment, and not via milk from their corresponding scrapie-affected ewes. Considering the minimal amount of PrP^d ^found in the distal ileum of the lambs culled early, it is unlikely that shedding of large amounts of PrP^d ^occurred between partner lambs at mixing. Assuming the lateral transmission controls were exposed to a high dose of infectivity when first introduced to the scrapie milk recipients and that the infectivity continued to build-up in the intervening 150 days, the severity of labelling should have been at least similar to that of the scrapie milk recipients at their RAMALT examination. This was, however, not the case since three lambs had no detectable PrP^d ^and two lambs were PrP^d^-positive, with TBM labelling only, and only up to half the follicles were positive. The accumulation pattern is similar for all gut-associated lymphoid tissue, including Peyer's patches and RAMALT, as well as lymph nodes: typically, TBMs are the first cells to become positive, followed by FDCs [1,2,13]. This suggests that the lateral transmission controls are at an earlier stage of infection than the scrapie milk recipients at the same time after mixing. Scrapie milk recipients were mixed at a median age of 16 days, while lateral transmission controls were introduced at a median age of 72 days. Age susceptibility to scrapie infection has been demonstrated, and a study based on data from a scrapie flock observed over a ten-year period has indicated that the risk of scrapie infection in sheep is associated with development of Peyer's patches and is highest in the first year of life [14]. The density of lymphoid follicles in Peyer's patches is greatest within the first three months of life in Cheviot sheep [15]. In sheep, involution of Peyer's patches begins at around twelve weeks of age, and is generally complete by 18 months of age [16]. Therefore it likely that, when first exposed to the environment, at least three of the lateral transmission controls (ten weeks old when mixed) had a similar number of Peyer's patches to the scrapie milk recipients and so were equally susceptible to infection. From the severity of labelling in the RAMALT samples it appears that the majority of the scrapie milk recipients were infected via milk. The single scrapie milk recipient (lamb 07–1357, see table 1) with TBM labelling only may have been infected via lateral transmission. However, weak or absent labelling in the lateral transmission controls suggests that the level of infectivity at mixing was probably not sufficient for lateral transmission to occur immediately at mixing. This is supported by the absence of PrP^d ^in any of the examined gut-associated lymphoid tissues of a culled scrapie milk recipient that was already mixed with others at eight days of age and culled at 105 days of age (lamb 518/7). Therefore the lamb with the weak labelling was probably infected via milk, but with a lower titre of infectivity.

Lateral transmission of scrapie in the absence of ewes, which may shed the scrapie agent around parturition, has been demonstrated. VRQ/VRQ sheep, which had been born to TSE-free ewes introduced to a flock with a high incidence of scrapie resulting in potentially high environmental contamination, had detectable PrP^d ^in tonsils at approximately 19 months of age, although maternal transmission could not be completely excluded [17]. It was thus unexpected to find PrP^d ^in two of five lateral transmission controls already at approximately eight months after mixing since they were housed in a previously scrapie-free environment and only had contact with the scrapie milk recipients, which were supposed to be in the early, pre-clinical stage of infection. Exposure to saliva (for example, by sharing food and water trough), faeces or urine, which have shown no detectable scrapie infectivity in mouse or goat transmission studies [6], was most likely responsible for infection of the controls, and it has to be seen whether they will develop clinical disease with a similar incubation period as the scrapie milk recipients.

The results from this study indicate that the risk of the transmission of scrapie via milk may be higher than previously thought. As a full lactation was fed to the lambs we are unable to determine if colostrum, which is secreted by the mammary gland in the first four days of lactation, contains a high level of immunoglobulins and is considered unfit for human consumption, or subsequent milk, or both, carry infectivity. Milk collected at any time during the lactation may be potentially infectious. A recent study in scrapie-affected sheep with concurrent lymphofollicular mastitis caused by the Maedi-Visna virus demonstrated the presence of PrP^d ^in mammary glands [18]. The authors hypothesised that milk from these cases could potentially serve as a vehicle for transmission of scrapie, although PrP^d ^has not yet been detected in milk itself. Mastitis produces an increased SCC in milk and may result in milk carrying more infectivity, since it has been shown that TSE can be transmitted in sheep by transfusion of blood and blood cells [19,20]. We found PrP^d ^in the lymphoid aggregates of the mammary gland of the ewe (1010/6) that provided the milk for one PrP^d^-positive lamb. Nine scrapie-affected ewes, including the only ewe (number 139/6) that fed a lamb that was without detectable PrP^d ^in its lymphoreticular tissues, had somatic cell counts of above 100,000 cells/ml (considered to be the threshold level for subclinical mastitis in ewes [21]) at some stage in their lactation (see Table 3), although other physical signs of mastitis were absent. The milk was macroscopically unremarkable at that time hence a bacteriological examination of the milk was not conducted. Other studies have shown that the SCC can exceed 1,000,000 cells per ml milk in some ewes without evidence of infection of the mammary gland [22-24]. Indeed, a bacteriological examination of the milk in another scrapie-affected ewe that is currently being milked and also presented with a SCC of 161,000 cells and 1,630,000 cells per ml on two subsequent weeks did not show any bacterial growth (T Konold, unpublished observation). Ewe 1010/6 had a SCC above 1,000,000 cells/ml on three subsequent weekly examinations, and a subclinical mastitis may have been present. Bacteria associated with subclinical mastitis are primarily coagulase-negative staphylococci, various streptococci and occasionally *Staphylococcus aureus *and *Mannheimia *spp. [25]. The SCC is also dependent on the stage of lactation and the time of milk collection [26]. It is usually higher towards the end of the lactation, and two ewes had indeed the highest SCC at the last sample. There is usually an increase in the morning, and samples for SCC in our study were taken in the morning. Another factor that affects SCC is the milking technique. It is lower in milk of hand milked or machine milked ewes compared to ewes with suckling lambs [23]. This would imply that lambs that suckled naturally would consume milk with an even higher SCC than in our study.

PrP^d ^was not detectable in the third culled lamb (number 518/7) fed milk from the scrapie-affected ewe 139/6. As mentioned earlier, involution of Peyer's patches and subsequent reduction of lymphoid follicles takes place from about 84 days of age in lambs [16], which may explain why there were less visible follicles in the examined sections from the 105 day-old lamb compared to the younger lambs, and, given that the two PrP^d^-positive, culled lambs presented with only a few PrP^d^-positive follicles out of the total examined, it is possible that follicles with detectable PrP^d ^may have been missed in this lamb. However, other lymphoid tissues, such as the mesenteric lymph nodes or the spleen, have been shown to contain PrP^d ^from two months of age in naturally infected VRQ/VRQ sheep [1,2]. The absence of PrP^d ^in the mesenteric lymph nodes and the spleen of the 105 day-old lamb thus suggests that the milk from the scrapie-affected ewe 139/6 did not carry enough infectivity to achieve PrP^d ^accumulation in the lymphoid tissues, either because PrP^d ^was not secreted via the milk at all or the amount of PrP^d ^was not sufficient enough to accumulate in the lamb. The ewe's milk presented with a SCC as high as others but the length of lactation was shorter compared to most of the other ewes, although ewe number 140/6 also had a short lactation but fed a lamb that accumulated PrP^d ^in RAMALT. However, this ewe's milk was discoloured and it is likely that blood cells rather than somatic cells present in the milk may have caused transmission. Unfortunately, the amount of collected milk in ewe 139/6 was too small to feed two lambs to confirm the absence of PrP^d ^in RAMALT of a partner lamb.

We have demonstrated that scrapie can be transmitted to genetically susceptible lambs via milk. This model may or may not be applicable to scrapie in sheep of other genotypes, or to other species infected with other prion strains where the pathogenesis of prion infection may be different. For example, in scrapie cases of particular genotypes [27] and BSE in cattle [28,29], the distribution of PrP^d ^is largely restricted to the gut-associated lymphatic tissue and nervous system, so prions may not be secreted in the milk. The lack of published information about infectivity of sheep milk has made it difficult to estimate the human health risk associated with milk from sheep and goats should BSE be found in small ruminants [30]. It is inevitable that should infectivity be present in the milk of BSE-infected sheep, the risk to humans would be particularly dependent on the prevalence of BSE infection in sheep in the national flock. This is acknowledged to be close to zero at present [31], but little is known about historical prevalence. The demonstration of probable natural transmission of BSE between mother and lamb in an experimentally infected flock [32] does however raise the issue of the possible routes of transmission for BSE in the perinatal period.

## Conclusion

These results indicate that there is a risk of the transmission of scrapie from ewe to lamb via milk or colostrum. Infection of lambs via milk may result in shedding of the infectious agent into the environment as we also observed infection of other lambs raised using scrapie-free ewes and then mixed in with the scrapie milk recipients. The study continues with the remaining lambs kept alive to assess if clinical disease develops, and further milk collection and feeding is planned to improve our estimate of the risk of transmission.

## Methods

All procedures involving animals were approved by the Home Office under the Animals (Scientific Procedures) Act 1986.

### Scrapie-affected ewes

Milk was collected by hand from twelve ewes (nine Poll Dorset, two Poll Dorset × Friesland crossbreds and one Cheviot sheep) throughout their lactations. Weekly milk samples were collected after the first week of lactation (colostral period), and the SCC was determined (National Milk Records plc, Chippenham, UK). Milk samples from individual ewes were stored at below -20°C for use in the following year. The ewes were kept in a flock with a high incidence of scrapie [17] and had PrP genotypes associated with a high risk of developing scrapie (V_136_R_154_Q_171 _homozygotes as determined by genotyping the PrP gene at codons 136, 154 and 171 [33]). The TSE status of each ewe was determined by immunohistochemical examination of a sample of RAMALT collected from the rectum under local anaesthesia (mixture of Lidocaine and Prilocaine, AstraZeneca, London, UK) prior to milking and examined with the monoclonal antibody (Mab) R145 [34], except for two ewes that had already shown signs of scrapie at that time. The RAMALT biopsy was preferred to a tonsil biopsy because the test sensitivity is similar in both tissues [34,35] and it has no known side-effects compared to a tonsil biopsy, which requires general anaesthesia.

All ewes were culled after developing definite clinical signs of scrapie, which were a combination of at least two of the signs 'changed behaviour (nervousness)', 'intense pruritus (rubbing)', 'wool loss or skin lesions', 'positive scratch test' [36], 'head tremor', 'ataxia' and 'loss of body condition'. Scrapie was confirmed by immunohistochemical examination of a formalin-fixed sample of the brainstem (obex) with Mab R145 [37] as well as Western immunoblot of a proteinase-digested sample of the caudal medulla with Mabs 6H4 and P4 (VLA Hybrid technique [38]). Various samples from peripheral lymph nodes, organs and glands were collected and formalin-fixed or frozen at -80°C but, for the purpose of this report, a section of the fixed samples of mammary gland and its regional lymph node, the superficial inguinal (supramammary) lymph node, from the ewes that supplied the milk for the three lambs culled early in the study were stained with haematoxylin-eosin (H&E) and immunolabelled with Mab R145 for histopathological and immunohistochemical examination respectively. The H&E section of the mammary gland from each animal was examined and the total number of lymphoid aggregates counted. A serial section was immunolabelled and examined to identify lymphoid aggregates corresponding to those seen on the H&E section. The number of positive and negative aggregates was recorded. A positive lymphoid aggregate was defined as an aggregate with disease-specific immunolabelling of a morphology similar to that seen in published images of immunolabelling in the mammary gland [18] and other mucosal-associated lymphoid tissues. A negative lymphoid aggregate was defined as an aggregate without disease-specific immunolabelling.

### Recipient lambs (lambs fed milk from scrapie-affected ewes)

Recipient sheep were 18 VRQ/VRQ Cheviot lambs born from ewes that were the offspring of ewes imported from New Zealand (NZ), which is free from scrapie, and that were kept in a flock that had never had any cases of classical scrapie. This flock was established to provide TSE-free sheep for research projects and is maintained under strict biosecurity controls to prevent the occurrence of scrapie. The TSE-free status of the dams was confirmed by immunohistochemical examination of a section of the obex (see above) and Bio-Rad Platelia ELISA (Bio-Rad Laboratories Ltd., Hemel Hempstead, UK) on a proteinase-digested sample of the caudal medulla according to the manufacturer's instructions [33]. Lambs were housed in medium security accommodation that had never housed any TSE-affected animals before. Each pen was decontaminated prior to housing of sheep with sodium hypochlorite (20% solution with 20,000 ppm available chlorine, which is effective against prions [39]). The milk was thawed and fed to the lambs in the same order it had been collected. If the daily amount of milk collected on any day was not sufficient to satisfy the appetite of the lambs, milk collected on the following day was fed afterwards. Milk from a single ewe was used to feed one or two lambs depending on the amount available. Each 'set' of lambs was housed in a separate pen to ensure that the lambs only received milk from the same single ewe. After all milk had been consumed, the lamb sets were mixed and received milk replacer (Lamlac, Volac International Ltd., Royston, UK), which did not contain any animal-derived product except for bovine whey protein. They were weaned at approximately six weeks of age, when they were provided with water and hay *ad libitum *and a daily ration of concentrate feed. Mixing was conducted in stages due to the various amount of milk that was fed to the lambs (see Table 1).

### Controls

"Building controls" to control for environmental contamination comprised ten VRQ/VRQ Cheviot lambs that were born from ewes from the same NZ-derived flock (TSE-free status confirmed by post-mortem test as detailed above) and housed with their dams in the same building at around the same time as the recipient lambs but in separate pens. They were reared naturally on their dam and weaned at approximately eight weeks of age.

It was acknowledged from the outset of the experiment that scrapie milk recipients had to be mixed because the number of available pens did not allow housing of small groups for a longer time period. In addition, sheep should be kept in groups to prevent distress caused by social isolation [40], and additional newborn lambs were not available to be housed alongside the single lambs that were fed milk from an individual scrapie-affected ewe. In order to investigate whether lateral transmission could occur after mixing, five Cheviot lambs from the NZ-derived flock were introduced to the weaned recipient lambs as "lateral transmission controls". Due to late availability of these lambs, they were mixed between one and three months after mixing of the milk recipient lambs (see Table 1).

Weaned control lambs received the same food as the weaned recipient lambs.

### Postmortem examination of lambs

Samples from lymphoreticular tissues (distal ileum with Peyer's patches, mesenteric lymph node and spleen, which present with PrP^d ^accumulation even in the very early stages of scrapie [1,2]) and the brain were collected from lambs culled due to disease and one half formalin-fixed and the other half frozen at -80°C. The distal ileum was rolled up for fixation and embedded in wax as such for further processing to increase the amount of lymphoid follicles in the section. Immunohistochemical examination was performed on the fixed lymphoreticular tissue [34] and the obex; a fresh sample of the caudal medulla was additionally examined by WB (VLA Hybrid technique, see above).

### Live animal sampling in lambs

A RAMALT sample was taken from each lamb at approximately seven months of age and examined by immunohistochemistry as described above. A second RAMALT biopsy was taken and examined similarly from all control lambs approximately three months later, which was equivalent to the maximum delay between mixing of scrapie milk recipients and introduction of lateral transmission controls.

### Microscopic images

The images were captured using the microscope Eclipse E400 with attached digital camera DXM1200 (Nikon UK Ltd., Kingston, UK) and the image-processing and analysis software Lucia G, Version 4.82, (Laboratory Imaging, Prague, Czech Republic). Photoshop Elements 5 (Adobe Systems Europe Ltd., Uxbridge, UK) was used for image cropping and text embedding.

## Authors' contributions

TK proposed the study with the help of SJB and HAS, wrote the manuscript and was the overall project manager. The projects supplying the sheep were managed by SJB and HAS. SJM performed the immunohistochemical examinations. All authors read and approved the final manuscript.
